# Classical and Alternative Activation and Metalloproteinase Expression Occurs in Foam Cell Macrophages in Male and Female ApoE Null Mice in the Absence of T and B Lymphocytes

**DOI:** 10.3389/fimmu.2014.00537

**Published:** 2014-10-28

**Authors:** Elaine Mo Hayes, Aikaterini Tsaousi, Karina Di Gregoli, S. Rhiannon Jenkinson, Andrew R. Bond, Jason L. Johnson, Laura Bevan, Anita C. Thomas, Andrew C. Newby

**Affiliations:** ^1^Bristol Heart Institute, School of Clinical Sciences, University of Bristol, Bristol, UK

**Keywords:** atherosclerosis, macrophages, lymphocytes, cytokines, plaque rupture

## Abstract

**Background:** Rupture of advanced atherosclerotic plaques accounts for most life-threatening myocardial infarctions. Classical (M1) and alternative (M2) macrophage activation could promote atherosclerotic plaque progression and rupture by increasing production of proteases, including matrix metalloproteinases (MMPs). Lymphocyte-derived cytokines may be essential for generating M1 and M2 phenotypes in plaques, although this has not been rigorously tested until now.

**Methods and results:** We validated the expression of M1 markers (iNOS and COX-2) and M2 markers (arginase-1, Ym-1, and CD206) and then measured MMP mRNA levels in mouse macrophages during classical and alternative activation *in vitro*. We then compared mRNA expression of these genes *ex vivo* in foam cells from subcutaneous granulomas in fat-fed immune-competent ApoE knockout (KO) and immune-compromised ApoE/Rag-1 double-KO mice, which lack all T and B cells. Furthermore, we performed immunohistochemistry in subcutaneous granulomas and in aortic root and brachiocephalic artery atherosclerotic plaques to measure the extent of M1/M2 marker and MMP protein expression *in vivo*. Classical activation of mouse macrophages with bacterial lipopolysaccharide *in vitro* increased MMPs-13, -14, and -25 but decreased MMP-19 and TIMP-2 mRNA expressions. Alternative activation with IL-4 increased MMP-19 expression. Foam cells in subcutaneous granulomas expressed all M1/M2 markers and MMPs at *ex vivo* mRNA and *in vivo* protein levels, irrespective of Rag-1 genotype. There were also similar percentages of foam cell macrophages (FCMs) carrying M1/M2 markers and MMPs in atherosclerotic plaques from ApoE KO and ApoE/Rag-1 double-KO mice.

**Conclusion:** Classical and alternative activation leads to distinct MMP expression patterns in mouse macrophages *in vitro*. M1 and M2 polarization *in vivo* occurs in the absence of T and B lymphocytes in either granuloma or plaque FCMs.

## Introduction

Atherosclerotic plaque rupture underlies most myocardial infarctions and thromboembolic strokes, which are principal causes of mortality and morbidity worldwide (https://apps.who.int/infobase/mortality.aspx). Macrophages play a key role in atherosclerosis progression, as demonstrated by their abundance in human plaques as foam cells and by the dramatic reduction in atherosclerosis in mice after genetic (1) or pharmacological (2) deletion of macrophages. Furthermore, production of mediators from activated macrophages is believed to be important in making plaques vulnerable to rupture (3). Reactive oxygen species, cytokines, and cell surface modifying proteinases produced by macrophages promote apoptosis, thereby contributing to the cellular rarefaction of vulnerable plaques (4). Moreover, extracellular proteinases, in particular matrix metalloproteinases (MMPs) can directly degrade the extracellular matrix and promote plaque instability (5). These proposed mechanisms are supported by the histological appearance of vulnerable plaques, which contain an abundance of macrophages expressing MMPs but a relative lack of smooth muscle cells (SMC) and extracellular matrix proteins, importantly collagens, which contribute tensile strength to the plaque cap (5). Intervening to diminish the production of these harmful mediators is therefore a rational approach to new therapies and this motivates efforts to understand the cellular and molecular mechanisms involved.

Early work highlighted the functional diversity of macrophages (6) and the existence of distinct phenotypes has become increasingly discussed (7). Polarization of macrophage into a so-called classically activated or M1 phenotype was recognized, possibly reflecting the context of infection where pathogen associated molecular patterns (PAMPs) and pro-inflammatory lymphocyte-derived cytokines, particularly interferons (IFNs) and interleukins (ILs) occurred in the same microenvironments. Consistent with this, the M1 phenotype is simulated *in vitro* by the combined action of PAMPs acting through Toll-like receptors (TLRs) and IFNγ (8), with some evidence for synergy. Mechanisms underlying synergy include the ability of IFNγ to prime responses to PAMPs by inducing expression of TLRs and their co-activators (9). Synergy also results from the combined activation of differing signaling pathways for TLRs through nuclear factor-κB (NF-κB) (9) and IFNγ through signal transducer and activator of transcription (STAT-1) (10).

The actions of IFNγ have led to the hypothesis that Thelper1 (Th1)-lymphocytes may be essential for, or at least prominent contributors to, M1 polarization *in vivo*, a concept that has been acknowledged in papers dealing with atherosclerosis (11–13). It is consistent with studies showing that knockout (KO) of IFNγ and its receptors reduces atherosclerosis progression (3), although the impact on M1 polarization was not measured directly in any of these IFNγ KO investigations. On the other hand, it is well recognized that other cytokines and combinations of cytokines that activate the pathways leading to M1 states could bypass the requirement for IFNγ (9, 14). Conversely, macrophages can polarized to a variety of alternatively activated or M2 states. The so-called M2a phenotype occurs after treatment with IL-4 or IL-13, which are potentially produced by Thelper2 (Th2) lymphocytes (15). Overall, this leads to the idea that T-lymphocytes, and, by implication, adaptive immunity play an essential part in M1/M2 macrophage polarization in atherosclerotic plaques. On the other hand, data from other areas of inflammation support the conclusion that M1/M2 polarization is primarily a function of innate immunity and that lymphocytes play a minor, at best modulatory role (16). Resolving this controversy is important for human atherosclerosis since a variety of immunotherapies have been proposed and some have already entered clinical trials (17).

Our present aim was formally to test the requirement for T and B lymphocytes in M1 and M2 macrophage activation and MMP production using an established ApoE-KO mouse model of atherosclerosis formation. To do this, we compared the expression of M1 and M2 markers, MMPs and the endogenous tissue inhibitors of MMPs (TIMPs) in foam cell macrophages (FCMs) from subcutaneous granulomas and atherosclerotic plaques in ApoE KO and ApoE/Rag-1 double-knockout (DKO) mice, which lack all T and B lymphocytes. As it is already known that there are differences in inflammation and plaque development between genders (18, 19), we used both male and female mice in this study. The mice were fed on a high-fat diet because it was previously shown that these conditions produced similar sized plaques in both genotypes (20). Hence, we could be sure that measurements of the prevalence of macrophages phenotypes would not be an artifact of different stages of atherosclerosis progression or plaque sizes.

## Materials and Methods

### Bone marrow monocyte isolation and differentiation to bone marrow-derived macrophages

Mouse femurs and tibias were excised from C57BL/6 mice (Charles River, UK) on a normal diet. The bone ends were cut and bone marrow was flushed out with sterile phosphate-buffered saline (PBS). Erythrocytes were removed using ACK lysis buffer (Life Technologies, UK) and the pelleted white blood cells resuspended in PBS and counted, giving an average yield of 40 × 10^6^ cells per mouse. Cells were plated into 24-well plates at 0.5 × 10^6^ cells/well and grown in RPMI 1640 media (Life Technologies) supplemented with antibiotics, glutamine, and 20% fetal calf serum (FCS) (Life Technologies) in the presence of 20 ng/ml recombinant human macrophage colony-stimulating factor (M-CSF, R&D Systems, USA). The media was changed every 3–4 days. After 7–10 days, when the cells had differentiated into macrophages, M-CSF was removed and the cells exposed to selected cytokines for 18 h in serum-free media (SFM), as specified in the text. These included mouse (m) IFNγ at 20 ng/ml (Miltenyi Biotec, USA), human (h) tissue necrosis factor (TNF)-α at 10 ng/ml (R&D Systems, USA), mIL-4 at 10 ng/ml (PreproTech, UK), and lipopolysaccharide (LPS) at 10 ng/ml (Sigma-Aldrich, USA, L2654). Human cytokines were used only when their efficacy on mouse cells had been previously documented by the suppliers.

### Peripheral blood mononuclear cell isolation and differentiation

Pooled peripheral blood was collected by cardiac puncture from C57BL/6 mice with heparin as anti-coagulant. Monocytes were then isolated density gradient separation on Ficoll Paque Plus and differentiated to macrophages as previously described (21).

### RNA extraction, reverse transcription, and quantitative PCR

RNA lysates from cultured macrophages were collected using RLT solution (Qiagen Ltd, UK) with β-mercaptoethanol and the total RNA extracted using the Qiagen RNeasy kit (Qiagen Ltd), according to the manufacturer’s instructions. The quantity and quality of resulting RNA was assessed using a NanoDrop ND-1000 spectrophotometer (LabTech International, UK). Samples of cDNA were generated using QuantiTect Reverse Transcription Kit (Qiagen Ltd), according to the manufacturer’s instructions and the resulting cDNA was diluted 1:1 in 10 mM TrisHCl, pH 8.0. Real time quantitative PCR was performed in a Roche Light Cycler 1.5 (Roche, UK) to quantify the steady-state concentration of RNA, using the QuantiTect SYBR Green PCR Kit (Qiagen Ltd). The primers used are listed in Table 1. Each reaction contained 2.5–7 ng RNA and 0.5 μM primers. Initial denaturation (15 min at 95°C) was followed by 55 cycles of denaturation (15 s at 95°C), annealing (20 s at 60°C), and extension (25 s at 72°C). Copy numbers of gene transcripts per total nanogram RNA input were calculated using standard curves constructed as recommended by from purified amplicon (Bioline, USA).

**Table 1:** **Primer sequences used for quantitative RT-PCR**.

Gene	Primer sequence
ARG-1	AGTCTGGCAGTTGGAAGCATCTCT
	TTCCTTCAGGAGAAAGGACACAGG
COX-2	ATACTGGAAGCCGAGCACCTTTGG
	ATGGTGGCTGTTTTGGTAGGCTGT
CD206	CCATTTATCATTCCCTCAGCAAGC
	AAATGTCACTGGGGTTCCATCACT
FIZZ1	AGAGGTGGAGAACCCAGCTTTGAT
	TTTCAAGAAGCAGGGTAAATGGGCA
IL-12p35	CCACAACAAGAGGGAGCTGCCTGCCC
	AGTGCTGCGTTGATGGCCTGGAACT
IL-12p40	AGACCAGGCAGCTCGCAGCAAAGCA
	GACACATCCCACTCCCACGCTGCC
iNOS	CTCATGACATCGACCAGAAGCGT
	TATATTGCTGTGGCTCCCATGTTG
MMP-2	GGCTGACATCATGATCAACTTTGG
	GCCATCAGCCGTTCCCATACTTTAC
MMP-3	GCATCCCCTGATGTCCTCGTGG
	TCCCCGGAGGGTGCTGACTG
MMP-8	TGCCTCGATGTGGAGTGCCTGA
	GCCCTTGACAGCTGTGGCGT
MMP-9	AGAGAGGAGTCTGGGGTCTGGTTT
	GAGAACACCACCGAGCTATCCACT
MMP-12	AATTACACTCCGGACATGAAGCGT
	GGCTAGTGTACCACCTTTGCCATC
MMP-13	ATGATGATGAAACCTGGACAAGCA
	ATAGGGCTGGGTCACACTTCTCTG
MMP-14	ACCACAAGGACTTTGCCTCTGAAG
	CACCGAGCTGTGAGATTCCCTTGA
MMP-19	GATGAACTGGCCAGAACTGACCTT
	GTCCCCGGTTGATGAGTTAGTGTC
MMP-23	CAAGGTTGGTGAGAGAGGGTAGGA
	AGGAGTAGGTGCTGAGAACACGCT
MMP-25	CTCTGAGTGGCAGTGTTTGGAAGA
	TGATGTCAGGCTCCTGGTACTGAG
TIMP-1	AGGAACGAAATTTGCACATCAGT
	CAAAGTGACGGCTCTGGTAGTCCT
TIMP-2	GACTCCCCCTCAGACTCTCCCTAC
	CATATTGATACCACCGCACAGGAA
TIMP-3	CACATCAAGGTGCCATTCAGGTAG
	GTTCTCTCCTCCTCAACCCAAACA
Ym1	CAGGTCTGGCAATTCTTCTG
	GTCTTGCTCATGTGTGTAAGTG

### *In vivo* studies

Rag-1 KO mice that do not produce mature T or B cells (B6.129S7-*Rag-1^tm1Mom^*/J) and ApoE KO mice on a C57BL/6 background (B6.129P2-*Apoe^tm1Unc^*/J) were purchased from The Jackson Laboratories (USA), and bred together to create ApoE^±^/Rag-1^±^ mice. Breeding stocks of ApoE KO and ApoE/Rag-1 DKO mice were obtained by crossing the resulting F1 generation. Mice were kept in scantainers and given sterile food and water *ad libitum*. All animal work was in accordance with the Home Office Guidance on the operation of the Animals (Scientific Procedures) Act 1986 and conforms to the Guide for the Care and Use of Laboratory Animals published by the US National Institutes of Health (NIH Publication No. 85–23, revised 1996). Genotyping of the mice was performed on ear or tail pieces, using the Direct PCR kit (Bioquote, UK or Viagen Biotech, USA) after a Proteinase K digestion (Sigma, UK). PCR was performed using Crimson Taq Pol (New England Biolabs, UK), with dNTPs from Bioline (UK), using primers designed by The Jackson Laboratories. Male and female mice commenced a sterile high-fat diet (21–23% fat, Special Diet Services, UK) at 5 weeks of age, and were sacrificed with an anesthetic overdose 12 weeks later. Blood was taken via cardiac puncture and heparinized plasma was subsequently analyzed for total, HDL, and LDL/VLDL cholesterol (Cholesterol/Cholesterol Ester Quantification Kit, Abcam, UK), after a minor adaptation of the manufacturer’s instructions. The levels of selected M1 and M2 cytokines was assessed in additional samples of mouse plasma using a Bio-Plex Pro Mouse Cytokine Th1/Th2 Panel 8-plex (Bio-Rad, USA). After the cardiac puncture, fresh tissue samples were taken (tail tip, spleen, liver lobe) and the animals perfused via the heart with PBS, then 10% formalin, at a constant pressure of 100 mmHg, with outflow through the left jugular vein. The brachiocephalic artery (BCA) (with a small piece of aortic arch), heart and remaining ascending and descending aorta were cleaned and removed from each mouse. Other tissues harvested included thymus and remaining liver. Tissue blocks of spleen less than 0.5 cm thick were post-fixed in 10% formalin for 24 h for subsequent histological examination, as described below.

### Sponge implantation and foam cell macrophage isolation

ApoE KO or DKO mice were fed the same high-fat diet as above from 6 weeks of age. Two weeks later these mice had 0.5 cm^3^ sterile polyurethane sponges containing ~50 μl of Matrigel (VWR, UK) placed under the dorsal skin under halothane anesthesia to generate FCMs as described previously (22, 23). The mice were fed the high-fat diet for a further 4 weeks. Recovered sponges were either fixed and embedded for immunohistological examinations as described below or the FCM were isolated and studied *ex vivo*. Fresh sponges were treated with 0.75 ml undiluted Dispase (VWR, UK or BD Biosciences, USA) and then squeezed to obtain a cellular exudate. FCM were then purified, as previously described (22, 24) by flotation after centrifugation on a metrizamide gradient (1.3507 refractive index, Sigma) followed by differential adherence. Only foam cells (validated by Oil-red-O staining) float because of the relatively low buoyant density of lipid.

Samples were taken from each preparation immediately for protein or RNA isolation, and mRNA levels quantified as described above. Other cell preparations were cultured for a short period, to allow adherence to coverslips. Oil-Red-O (2% Oil-red-O in isopropanol; Sigma) staining was performed to confirm lipid content, and immunocytochemistry performed to confirm cell purity. Cells were also assessed for their proliferative capacity [BrdU (Sigma) incorporation, 8 h pulse] or *in situ* zymography (25). In this assay, the gelatinolytic capacity of the macrophages isolated from the sponges was determined using the EnzChek gelatinase/collagenase assay kit (Invitrogen, USA). Controls included cells treated with EDTA, 1,10-phrenanthroline (Sigma) or GM6001 (Millipore, UK), to prevent MMP activity. Cells were fixed in paraformaldehyde and mounted in Vectorshield + DAPI (Vector Labs, USA). Several fields were photographed on each coverslip and the proportion of cells with gelatinase activity as indicated by the loss of fluorescence of the DQ-gelatin substrate determined.

### Histological methods

The proximal aorta and BCA from each mouse were embedded in paraffin and 3 μm sections cut at 3 μm intervals from the atherosclerosis-prone areas of these vascular beds, as described previously (23, 26). The first section after the bifurcation of the BCA from the aorta was cleared and rehydrated and then stained using Miller’s elastin/van Gieson (EVG) and plaque dimensions were measured using image analysis software (Image Pro, DataCell, Maidenhead, UK), as described previously (23). The aortic sinus from each mouse was treated and examined in a similar fashion, with the first leaflet section (from the aorta) stained using EVG, with subsequent image analysis being performed (26). For immunohistochemistry, 3 μm sections were brought to water and antigen retrieval performed using citrate buffer. Non-specific binding blocked with 10% goat serum (Sigma) in PBS. Primary antibodies for SMC (α-smooth muscle actin), macrophages [*Griffonia simplicifolia* Lectin II (GSL)], iNOS, COX-2, CD206, arg-1, Ym-1, MMP-12, MMP-13, MMP-14, and TIMP-3 (see Table 2) were added to the sections and incubated either overnight at 4°C or for 1 h at room temperature. After washing and further incubations with goat anti-rabbit-biotin (Dako or Sigma) and ExtrAvidin-HRP (Sigma) staining was visualized using 3,3’-diaminobenzidine (DAB, Sigma). A negative control where the primary antibody was replaced with the relevant species IgG at the same dilution was always included. The percentage of the plaque area stained with each cell-specific or phenotypic marker or MMP/TIMPs antibody was determined using the same image analysis software detailed above. The number of buried layers was assessed manually on sections stained with EVG and on sections using antibodies that recognize SMC. Paraffin-embedded sponge sections were treated similarly, and the presence of markers of macrophage activation examined. Oil-Red-O staining was performed *en face*, and the percentage of fatty deposits in each aorta was measured using NIH ImageJ v1.43.

**Table 2:** **Primary and secondary antibodies and *Griffonia simplicifolia* Lectin II**.

	Catalog number	Type	Supplier
**Primary**
GSL II	B1215	Lectin	Vector labs
α-Smooth muscle actin	M0851	M_Mab	Dako
arginase 1	sc-20150	Rb_PAb	Santa Cruz
BrdU	B2531	M_Mab	Sigma
COX2	ab15191	Rb_PAb	Abcam
iNOS	ab15323	Rb_PAb	Abcam
MOMA-2	ab33451	R_Mab	Abcam
MMP-12	ab52897	Rb_MAb	Abcam
MMP-13	ab39012	Rb_PAb	Abcam
MMP-14	ab51074	Rb_MAb	Abcam
STAT1p (phospho Y701)	ab30645	Rb_PAb	Abcam
STAT6p (phospho Tyr641)	06–937	Rb_PAb	Millipore
TIMP-3	Ab39206	Rb_PAb	Abcam
Ym1/2	01404	Rb_PAb	Stemcell Technologies
rabbit IgG negative control	I5006		Sigma
mouse IgG2a negative control	M5409		Sigma
Goat serum	G9023		Sigma
Rabbit serum	R9133		Sigma
**Secondary antibody**
GtαM	E0433	Biotinylated	Dako
GtαM	B6649	Biotinylated	Sigma
GtαRb	E0432	Biotinylated	Dako
GtαRb	B6649	Biotinylated	Sigma
RbαR	E0468	Biotinylated	Dako

### Statistical methods

All analyses were performed using GraphPad InStat v3.05 (GraphPad Software, Inc. USA) or SPSS v21 (IBM, USA) software. Data were checked for normality (Kolmogorov and Smirnov normality test), and logarithmic transformation of data performed if necessary. Regression analyses were performed using Pearson’s correlation co-efficient. Statistical analyses of data were performed using Students *t*-test, a Mann–Whitney *U*-test or 1- or 2-way ANOVAs, with the 1-way ANOVA followed by a Bonferroni or Tukey-Kramer post-test. Data are expressed as arithmetic mean ± SEM or geometric mean and 95% confidence limits, and statistical significance defined as *P* < 0.05.

## Results

### *In vitro* studies in mouse bone marrow macrophages

**Figure 1:**
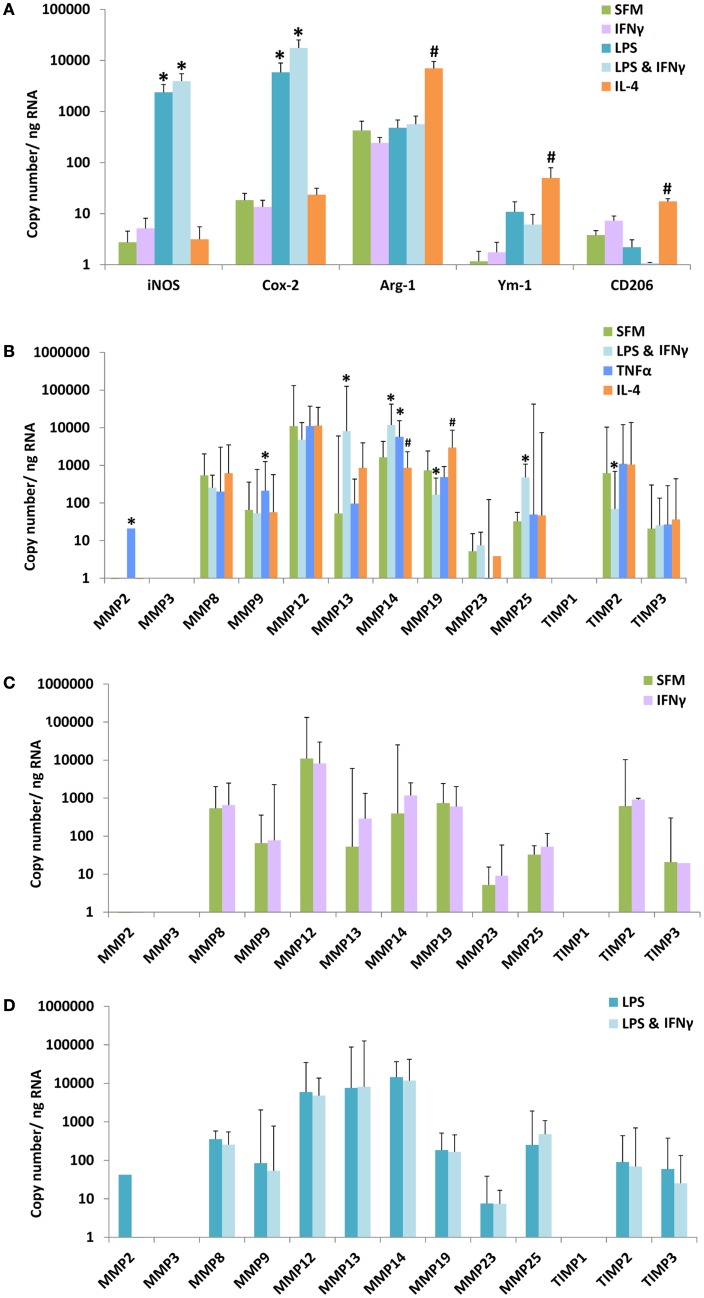
**Effect of M1/M2 polarization on mRNAs for markers, MMPs, and TIMPs in mouse bone marrow-derived macrophages**. Monocytes from mouse bone marrow were differentiated to macrophages in M-CSF and then treated with bacterial lipopolysaccharide (LPS), interferon-γ (IFNγ), tumor necrosis factor-α (TNFα), or interleukin-4 (IL-4) alone or in combination, as indicated. SFM = serum-free medium. **(A)** The effect of polarization by IFNγ, LPS, LPS + IFNγ, or IL-4 on M1/M2 markers (*n* = 4–5). **(B)** The effect of polarization by IFNγ + LPS, TNFα, or IL-4 on MMPs and TIMPs (*n* = 3–8). **(C)** Effect of IFNγ alone on macrophages incubated in SFM. **(D)** Effect of IFNγ on macrophages incubated in SFM containing LPS. Values are expressed as mean and SEM **(A)** or mean and 95% confidence intervals **(B–D)**. *M1 different from M0, ^#^M2 different from M0, *P* < 0.05.

Bone marrow proved a convenient source of large quantities of mouse monocytes that were converted to bone marrow-derived macrophages (BMDM) using M-CSF. BMDM were 97% F4/80 and CD11b double positive by flow cytometry (results not shown). We used mRNA expression for established M1 and M2 marker genes as positive controls for classic or alternatively activation. As expected from previous literature (15), classical activation with LPS alone or LPS plus IFNγ increased mRNA levels of inducible NO synthase (iNOS, NOS-2) and cyclooxygenase-2 (COX-2) (Figure 1A), whereas alternative activation with IL-4 increased mRNA expression of arginase-I (arg-1), Ym-1, and CD206. We then investigated the concomitant regulation of a wide spectrum of MMPs and TIMPs, many of which have been previously implicated in atherosclerosis (27). The most abundant mRNAs under unstimulated conditions were MMP-12 > MMP-8 = MMP-19 = MMP-14 = TIMP-2 > other MMPs and TIMPs (Figure 1B). Among the MMPs studied, MMP-13 showed the most dramatic 121-fold stimulation by LPS + IFNγ (Figure 1B, note the scale is logarithmic). LPS + IFNγ treatment also increased expressions of MMP-14 (11.3-fold) and MMP-25 (14.6-fold); and decreased expressions of MMP-19 (4.5-fold) and TIMP-2 (9.0-fold) (Figure 1B). Classical activation with a different mediator, tumor necrosis factorα (TNFα), significantly increased expressions of MMP-2 (77-fold), MMP-9 (3.5-fold), and MMP-14 (3.5-fold) but not of MMP-13 (Figure 1B). Classical activation with TNFα also increased MMP-9 and MMP-14 expression in blood derived macrophages, similar to BMDM, but did not affect any of the other MMPs or TIMPs (results not shown). Treatment with IFNγ did not increase mRNA levels of any MMP or TIMP in BMDM either alone or in the presence of LPS (Figures 1C,D). No effect of IFNγ was observed in blood derived macrophages either (results not shown). Alternative activation with IL-4 increased mRNA expression of only MMP-19 in BMDM (Figure 1B). These results revealed widely different levels of expression and divergent patterns of regulation of MMPs and TIMPs, which informed our choice of genes to measure in the subsequent *in vivo* experiments (see below).

### Circulating inflammatory cytokine and cholesterol levels in ApoE KO and ApoE/Rag-1 double-knockout mice

To investigate the impact of lymphocytes on M1/M2 polarization and MMP/TIMP expression, we compared ApoE KO and ApoE/Rag-1 DKO mice, which were genotyped using PCR to verify the deletion of ApoE alone or ApoE and Rag-1. The weight of the spleens of ApoE KO mice (115 ± 6 mg, *n* = 8) was reduced to (56 ± 9 mg, *n* = 10) in DKO mice and histological analysis of cut sections confirmed the absence of lymphocytes from DKO spleens (not shown). As expected, the expression of CD3 mRNA in the spleen, as a further marker of the presence of lymphocytes, was measurable in all ApoE KO mice tested (mean value 1.6 ± 0.5 copies/ng RNA, *n* = 10) but undetectable in all DKO mice tested. We used blood levels of cytokines related to Th1 and Th2 lymphocytes to further characterize the inflammatory status of ApoE KO and DKO mice. As in Table 3, circulating levels of IFNγ were low and not different between genotypes, whereas IL-4 levels were below the limits of detection of our assays. Of the more abundant cytokines, TNFα levels were not different, whereas GM-CSF, IL-12, and IL-10 levels were elevated (1.6, 2.2, and 2.5-fold, respectively) in female DKO compared to ApoE KO mice. The same trend was seen for IL-10 levels in male mice (Table 3). These results showed, unexpectedly, that cytokines associated with M1 polarization (i.e., GM-CSF and IL-12) and M2 polarization (IL-10) were at least the same or even elevated in DKO mice. We also noted that TNF-α levels were significantly 1.8-fold higher in male than female ApoE KO mice but not different in DKO mice (Table 3).

**Table 3:** **Concentration of inflammatory and anti-inflammatory cytokines in plasma from ApoE KO or ApoE/Rag1 KO mice**.

	Male ApoE KO		Male DKO		*P-*value (males)	Female ApoE KO		Female DKO		*P-*value (females)
	Mean	SEM	Mean	SEM		Mean	SEM	Mean	SEM	
IFNγ	11.88	4.353	5.680	2.902	0.2440	11.33	6.345	6.012	2.595	0.6275
TNFα	297.4^a^	56.00	206.3	23.16	0.1495	167.9	21.46	191.0	19.26	0.4331
GM-CSF	118.0	24.55	106.4	16.66	0.6959	72.85	11.24	113.6	13.22	**0.0304**
IL-12p70	133.6	38.79	129.9	37.60	0.9457	58.65	12.61	127.7	29.90	**0.0302**
IL-10	15.23	3.023	23.99	3.852	0.0964	10.32	3.0.03	25.38	2.486	**0.0012**

*Concentrations (picograms per milliliter) are expressed as mean and SEM, *n* = 9–10. Bold type indicates *P* < 0.05 for ApoE KO vs. DKO*.

*^a^Indicates significant gender difference for that genotype*.

Plasma cholesterol levels play a fundamental role in determining the extent of atherosclerosis in human beings and animal models. As shown in Table 4, the plasma concentrations of total cholesterol were not different between ApoE KO and DKO mice. HDL cholesterol was significantly increased sixfold in female DKO vs. ApoE KO mice and there was the same trend (1.9-fold) in the male mice. Conversely, LDL plus VLDL concentrations were significantly 1.8-fold lower in male DKO than ApoE KO mice and same trend (1.4-fold) was seen for the females. We noted that total cholesterol levels were significantly higher in males than females of either genotype. Furthermore, VLDL + LDL levels were significantly higher in the male than female ApoE KO mice (Table 4). Based on increased HDL and decreased VLDL + LDL, DKO mice of either gender might be expected to be protected from atherosclerosis compared with ApoE KO mice.

**Table 4:** **Concentration of cholesterol-containing lipoproteins in plasma from ApoE KO or ApoE/Rag1 KO mice**.

	Male ApoE KO		Male DKO		*P-*value (males)	Female ApoE KO		Female DKO		*P-*value (females)
	Mean	SEM	Mean	SEM		Mean	SEM	Mean	SEM	
Total	1360^a^	116.5	1215^a^	47.04	0.3939	802.4	64.65	707.3	59.73	0.3050
HDL	58.64	32.99	108.0	21.20	0.2364	14.39	3.177	85.86	41.76	**0.0022**
LDL + VLDL	931.3^a^	71.77	508.4	99.79	**0.0063**	504.4	55.29	362.3	68.75	0.1384

*Concentrations (milligrams per deciliter) are expressed as mean and SEM, *n* = 6. Bold type indicates *P* < 0.05 for ApoE KO vs. DKO*.

*^a^Indicates significant gender difference for that genotype*.

### M1/M2 marker and MMP mRNA levels in granuloma FCMs from subcutaneous sponges

We sought a ready source of foam cells generated *in vivo* to investigate expression of the M1/M2 markers and MMPs and TIMPs measured in our *in vitro* study of non-foamy macrophages. Atherosclerotic plaques are small and difficult to disrupt but hypercholesterolemia promotes the accumulation of foam cells at several more accessible sites in human beings and mice. For example, foam cells accumulate in the peritoneum (28) or in granulomas that form in sterile sponges implanted subcutaneously into atherosclerosis-prone mice (23). In this study, FCMs were isolated from subcutaneous granulomas. They were purified based on their decreased buoyant density by flotation over a density gradient. The yield of foam cells from subcutaneous sponges implanted for 6 weeks into ApoE KO or DKO mice was 4.07 ± 0.61 × 10^6^ and 5.87 ± 1.21 × 10^6^ cells, respectively, and did not significantly vary between genders. Foam cells from ApoE KO mice were 95.5 ± 1.6% macrophages (using MOMA-2 as a marker). FCMs had detectable levels of the same M1 and M2 markers seen in non-foamy BMDM, irrespective of whether they came from ApoE KO or DKO mice (Table 5). We concluded that FCMs acquired M1 marker genes in the absence of T and B lymphocytes. Indeed, there was a trend toward higher levels of mRNA expression of M1 markers, iNOS and COX-2, in DKO animals, although this was not significant (Table 5). Given this somewhat surprising conclusion, we measured additional M1 markers, namely IL-12 p35 and p40 and SOCS3, which were also detectable and showed no significant difference between ApoE-KO and DKO mice (Table 5). The mRNAs for M2 markers arg-1, and Ym-1 were also expressed at similar levels in granuloma FCMs from ApoE KO or DKO mice (Table 5), whereas CD206 was slightly elevated in male DKO compared to ApoE KO mice. The data suggested that M2 polarization also occurred efficiently in the absence of lymphocytes. To confirm this, we added measurements of FIZZ1 and IL-10, which also showed no difference between ApoE KO and DKO FCMs (Table 5). We noted a few significant gender differences. The mRNA levels of the M1 marker, IL-12p40, and the M2 markers, CD206 and Ym-1, were approximately 50% lower in granuloma FCMs from male compared to female ApoE KO mice.

**Table 5:** **Characteristics of foam cell macrophages obtained from subcutaneous sponges**.

	Male KO		Male DKO		*P-*value (males)	Female ApoE		Female DKO		*P-*value (females)
	Mean	SEM	Mean	SEM		Mean	SEM	Mean	SEM	
COX2	915	135	2053	729	0.3692	931	134	1306	243	0.1981
iNOS	56	7	474	305	0.3793	69	13	331	242	0.3357
IL-12p35	1085	88	904	130	0.2451	1563	299	1154	276	0.3316
IL-12p40	6^a^	1	9	3	0.7304	13	2	11	4	0.6432
SOCS3	1797^a^	204	4117	1060	0.1191	2679	327	3038	587	0.5896
Arg-1	79646	5095	87511	7825	0.3953	87172	14259	90946	10539	0.8345
CD206	1317^a^	198	2150	285	**0.0224**	2420	212	2293	372	0.7707
FIZZ1	377	58	518	82	0.1640	365	65	594	136	0.2810
IL-10	10262	1115	10234^a^	780	0.9852	12984	2289	19852	4366	0.1853
Ym-1	35^a^	5	78	31	0.3311	103	20	124	29	0.5530
MMP2	387	77	614	136	0.1462	535	97	681	173	0.4586
MMP9	104^a^	18	217	63	0.0720	182	22	155	14	0.3537
MMP12	176264	11743	150381	15260	0.1860	215896	28916	160661	26809	0.1831
MMP13	43800	3821	33103	3990	0.0664	48821	8573	34538	4202	0.1770
MMP14	4888	570	5402	462	0.4963	5214	917	5856	942	0.6332
MMP19	8486	1684	7893	930	0.7723	8756	998	7814	1268	0.5699
MMP25	23	3	112	47	0.6049	27	6	84	42	0.9307
TIMP-1	1674^a^	237	2321	399	0.1625	2543	330	1771	401	0.1571
TIMP-2	6624^a^	533	6984	730	0.6884	10456	682	6455	606	**0.0008**
TIMP-3	1203^a^	160	2728	689	**0.0352**	2228	356	2452	586	0.7434

*FCMs were isolated by density gradient centrifugation and differential adhesion from sponges implanted under the skin of ApoE KO or ApoE/Rag-1 DKO mice, as indicated. Levels of mRNAs of M1/M2 markers and MMPs/TIMPs are expressed as copies/ng total RNA (mean and SEM, *n* = 7–13). Bold type indicates *P* < 0.05 for ApoE KO vs DKO*.

*^a^Indicates significant gender difference for that genotype*.

**Figure 2:**
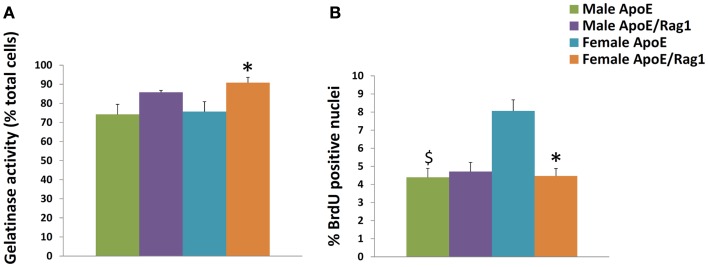
**Characteristics of foam cell macrophages (FCMs) obtained from subcutaneous granulomas**. Granuloma FCMs were isolated by flotation over a density gradient and differential adhesion from sponges implanted under the skin of ApoE KO or ApoE/Rag-1 DKO mice, as indicated. **(A)** *In situ* zymography (gelatinase activity, mean and SEM, *n* = 9–13). **(B)** Proliferative capacity (8 h BrdU pulse, mean and SEM, *n* = 8–14). *P < 0.05 vs. DKO, ^$^*P* < 0.05 indicates gender differences for that genotype.

The MMPs that were increased by classical activation in blood or BMDM, that is MMP-2, MMP-9, MMP-13, MMP-14, and MMP-25, were all expressed in granuloma FCMs, irrespective of Rag-1 genotype (Table 5). TIMP-2 (that was decreased by classical activation) and MMP-19 (that was decreased by classical activation and increased by alternative activation) were also expressed at high levels in both genotypes (Table 5). Female ApoE KO mice had 1.6-fold more TIMP-2 mRNA than the corresponding males. MMP-12 and TIMP-1 [that showed no relationship with classical/alternative phenotype under the conditions of our *in vitro* studies (Figure 1B)] were expressed irrespective of genotype, but TIMP-3 was significantly increased 2.3-fold in male DKO compared to ApoE KO mice. The proportion of granuloma FCMs able to degrade gelatin was determined using *in situ* zymography. A significantly higher percentage of granuloma FCMs had gelatinase activity from female DKO (91%) compared with ApoE KO animals (76%), and the same trend was evident in the in the males (86 vs. 74%) (Figure 2A), Pooling the data, the 88% of the DKO mice had gelatinase activity compared to 75% in the ApoE KO mice (*P* = 0.0026). The proliferative capacity of FCMs was assessed by measuring BrdU incorporation (Figure 2B). Proliferation was not different in male mice of either genotype but was increased almost twofold in female ApoE KO mice compared to DKO mice (*P* < 0.0001). Migration through a matrigel layer in a modified Boyden chamber assay (*n* = 3–5) was not significantly different amongst granuloma FCMs from the two genotypes (results not shown).

### M1/M2 marker protein expression and pathway activation in granuloma FCMs from subcutaneous sponges

**Figure 3:**
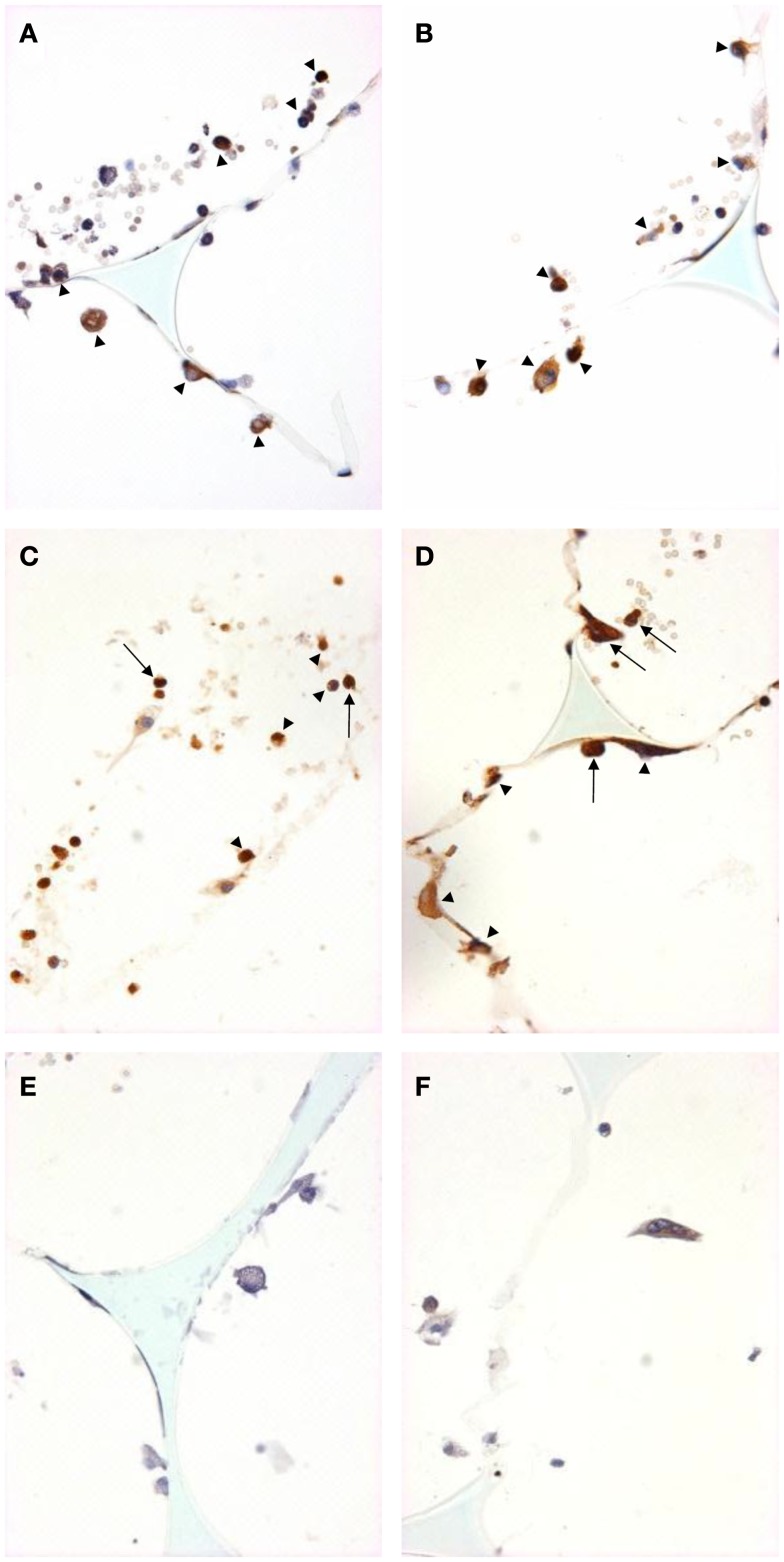
**M1 marker staining of sponge sections from ApoE KO or ApoE/Rag-1 DKO mice**. Sections from ApoE KO **(A,C,E)** or DKO mice **(B,D,F)** were stained to show immunolocalization of inducible nitric oxide synthase (iNOS) **(A,B)** or cyclooxygenase-2 (COX-2) **(C,D)**. Control sections **(E,F)** were exposed to non-immune IgG. Positive staining appears brown (DAB), nuclei blue-purple (hematoxylin), and sponge spicules light blue. Sections from either mouse strain could contain cells with positive cytoplasmic staining (arrowheads). Some cells did not stain, and some appeared to have COX-2 in their nucleus (arrows) (Magnification: ×600).

**Figure 4:**
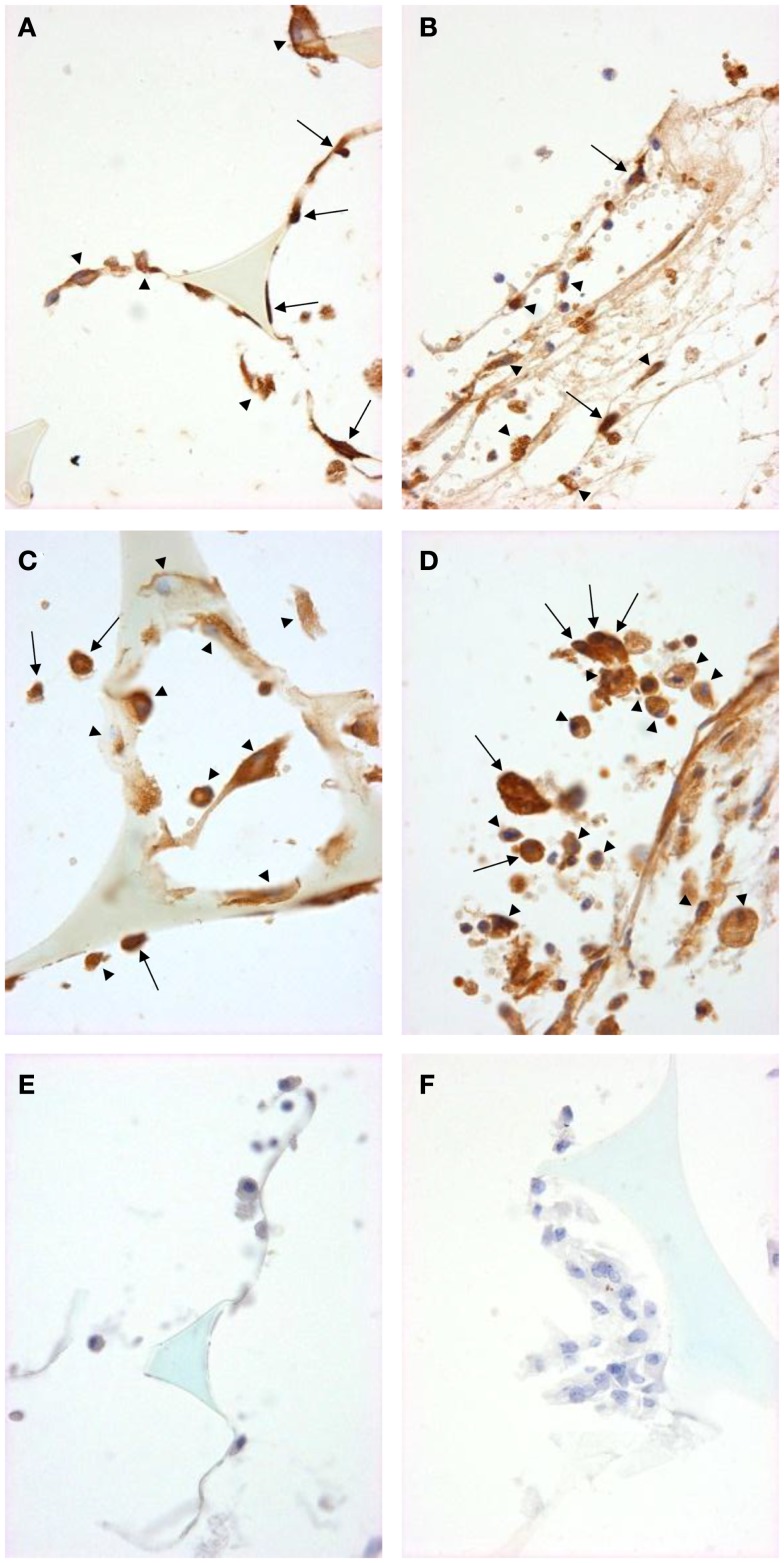
**Signal pathway activation in sponge granuloma sections from ApoE KO or ApoE/Rag-1 DKO mice**. Sections from ApoE KO **(A,C,E)** or DKO **(B,D,F)** mice were stained to show immunolocalization of NF-κB **(A,B)** or phosphorylated STAT1 **(C,D)**. Control sections **(E,F)** were exposed to non-immune IgG. Positive staining appears brown (DAB), nuclei blue-purple (hematoxylin), and sponge spicules light blue. Sections from either mouse strain contained many cells with positive cytoplasmic staining (arrowheads). Some cells did not contain detectable levels of NF-κB or phospho-STAT-1, while others appeared to have NF-κB or phospho-STAT-1 present in their nucleus (arrows) (Magnification: ×600).

**Figure 5:**
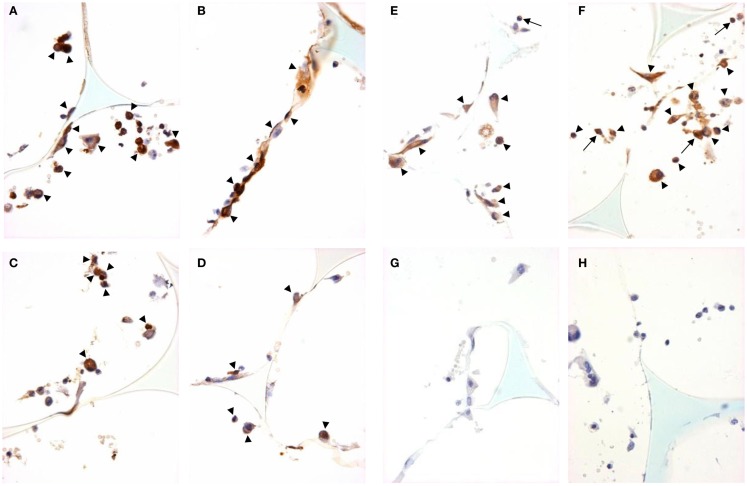
**M2 marker staining and signal pathway activity in sponge granuloma sections from ApoE KO or ApoE/Rag-1 DKO mice**. Sections from ApoE KO **(A,C,E)** or DKO **(B,D,F)** mice were stained to show immunolocalization of arginase-1 **(A,B)**, Ym-1 **(C,D)**, or phosphorylated STAT6 **(E,F)**. Control sections from ApoE KO **(G)** or DKO **(H)** mice were exposed to non-immune IgG. Positive staining appears brown (DAB), nuclei blue-purple (hematoxylin), and sponge spicules light blue. Sections from either mouse strain contained cells with positive cytoplasmic staining (arrowheads), but some cells did not contain detectible levels of arginase-1, Ym-1, or phospho-STAT-6. Some cells were observed with nuclear phospho-STAT-6 staining (arrows) (Magnification: ×600).

We used immunohistochemistry to confirm the protein expression of M1 and M2 markers *in vivo* by using sections taken from excised subcutaneous sponges. A subpopulation of granuloma cells in sponge sections stained for iNOS (Figures 3A,B) or COX-2 protein (Figures 3C,D) in either Rag-1 genotype. Control sections stained with isotype matched non-immune immunoglobulins had no staining (Figures 3E,F). Furthermore, a fraction of the granuloma cells in sponge sections stained for nuclear localized NF-κB (Figures 4A,B) or phosphorylated STAT-1 (Figures 4C,D), indicating that these cells had undergone activation of the signaling pathways that are associated with M1 activation. Sections stained with isotype matched non-immune immunoglobulins had no staining (Figures 4E,F). Some FCMs in sections also stained for arg-1 (Figures 5A,B), Ym-1 (Figures 5C,D), or phosphorylated STAT-6 (Figures 5E,F), demonstrating the presence of marker proteins and active signaling pathways that are associated with M2 macrophages. Sections stained with isotype matched non-immune immunoglobulins again had no staining (Figures 5G,H). Similar results to those with sponge sections were found by immunocytochemistry of FCM isolated from sponges (data not shown).

### Plaque size, composition, M1/M2 markers, and MMP protein expression in FCMs in atherosclerotic plaques from ApoE KO and DKO mice

**Figure 6:**
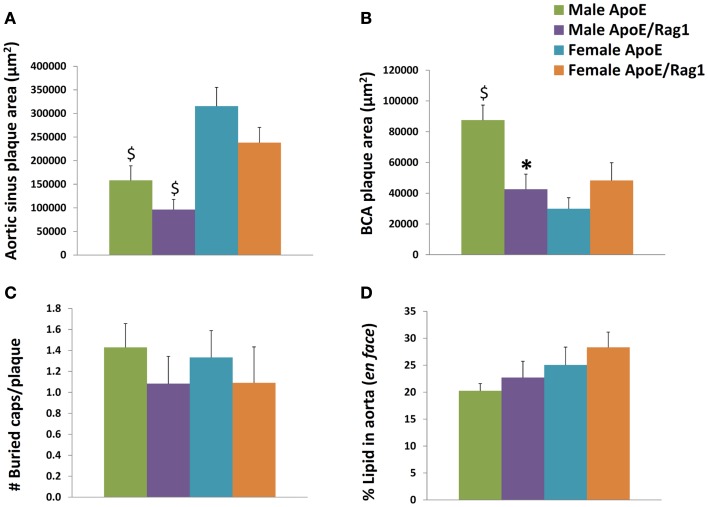
**Histological appearances of aortic sinus, brachiocephalic artery and aortic plaques in ApoE KO and ApoE/Rag-1 DKO mice**. **(A)** Area of plaque in the aortic sinus. The first section showing leaflet of the aortic valve was stained using Miller’s elastin/van Gieson (EVG) and the area of plaque in each section calculated using computer aided planimetry, *n* = 16–22. **(B)** A section taken 3 μm after the bifurcation of the brachiocephalic artery (BCA) from the aorta was treated and stained and examined in a similar fashion to A, *n* = 13–19. **(C)** The number of buried layers in plaque from BCA were assessed in EVG and α-SM-actin stained sections, *n* = 11–14. **(D)** Whole aorta were opened longitudinally and stained *en face* for the presence of neutral lipid using Oil-Red-O. The area of lipid-rich plaque in each aorta was calculated using computer aided planimetry, *n* = 10–11. Values are expressed mean and SEM. *P < 0.05 vs. DKO, ^$^*P* < 0.05 indicates gender differences for that genotype.

Plaques in the aortic sinus tended to be smaller in DKO compared with ApoE KO mice but this did not reach statistical significance (Figure 6A). However, in agreement with previous reports (18, 19), significant gender differences were observed (Figures 6A,B). Aortic sinus plaques were significantly bigger in females compared with males in ApoE KO (twofold: *P* = 0.0057) and DKO (2.5-fold: *P* = 0.0043) mice. Plaques in the BCA were 2.1 times smaller in the DKO compared to ApoE KO males (*P* = 0.004) but there was no significant difference between the two genotypes in the females (Figure 6B). Interestingly, ApoE KO males had 2.9 times larger plaques when compared with ApoE KO females (*P* < 0.0001) (Figure 6B), which was the opposite of the relationship observed in the aortic sinus. This difference has also been noted before (29). These differences reinforced our decision to stratify our data by gender.

Buried fibrous layers in BCA plaques have been suggested as a marker of plaque complexity or instability (30). However, we observed no linear regression between the number of buried layers and plaque size (*P-*values range from 0.20 to 0.68, *n* = 15–17) or macrophage content (*P-*values range from 0.29 to 0.99, *n* = 15–17), which are other measures that have been associated with plaque vulnerability. In any event, there were no significant differences in the number of buried layers in plaques from DKO compared to ApoE KO mice (Figure 6C). The area occupied by plaques in the aorta (as demonstrated by *en face* staining with Oil-Red-O) gives another measure of the extent of plaque progression in these mice. This did not change with phenotype or gender (Figure 6D).

The comparable plaque size in the aortic root of ApoE KO and DKO mice has been reported previously (20), and was consistent with the objective of our experimental design. It ensured that any difference in foam cell phenotype in the aortic sinus would be independent of plaque size. However, the larger plaque size in male BCA plaques in ApoE KO than DKO mice could complicate the interpretation of data relating to phenotypes in these mice.

**Figure 7:**
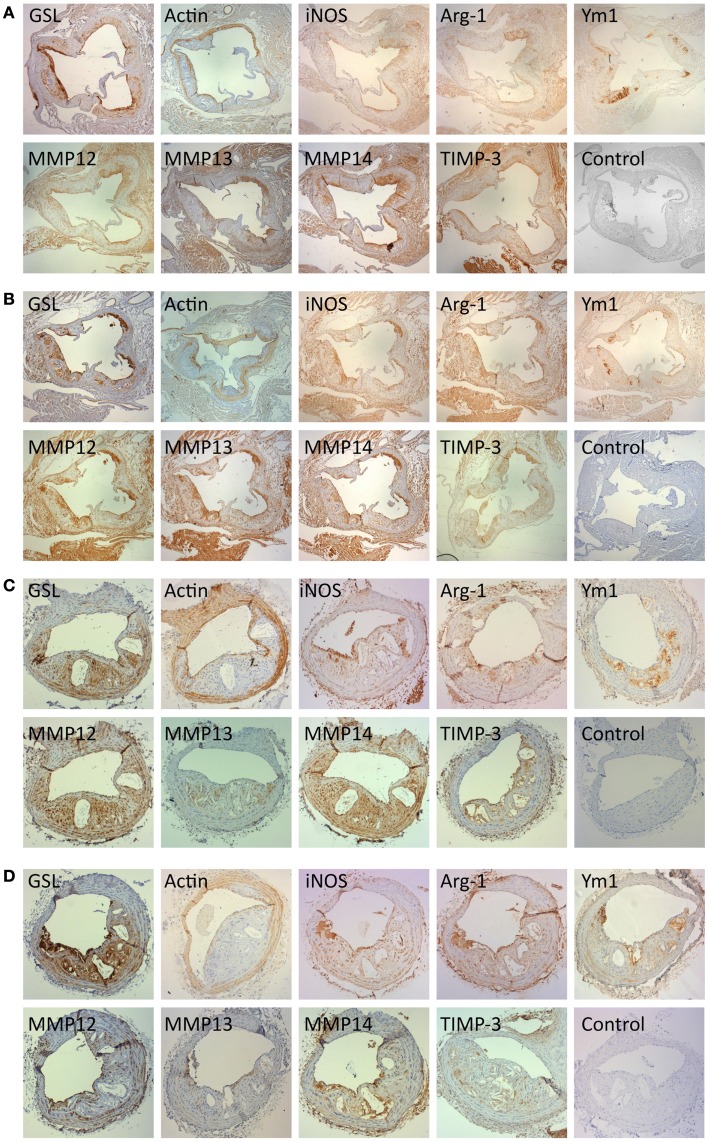
**Immunohistochemical staining for cell type, M1 and M2 markers and for MMPs and TIMPs**. Near consecutive sections to the section stained with elastin/van Gieson (EVG) were subjected to immunohistochemistry for macrophages [*Griffonia simplicifolia* lectin II (GSL)], smooth muscle cells (α-smooth muscle actin; actin), iNOS, arg-1, Ym-1, MMP-12, MMP-13, MMP-14, and TIMP-3, using the antibodies detailed in Table 2. Controls were performed with non-immune IgG or normal serum replacing the primary antibody. **(A)** Aortic sinus plaques from ApoE KO mice (Magnification: ×4). **(B)** Aortic sinus plaques from ApoE/Rag1 DKO mice (Magnification: ×4). **(C)** Brachiocephalic artery plaques from ApoE KO mice (Magnification: ×10). **(D)** Brachiocephalic artery plaques from ApoE/Rag1 DKO mice (Magnification: ×10).

Immunohistochemistry for α-smooth-muscle-cell-actin (α-SM-actin) was used to quantify the presence of vascular SMC and staining for GSL to quantify macrophages. As expected SMC were mainly found in the media and fibrous cap of plaques (Figures 7A–D). Most GSL positive had a foamy appearance on close examination and were therefore mainly FCMs (Figures 7A–D). However, some medial cells, presumably synthetic state SMC or SMC transdifferentiating toward macrophages (31) also stained with GSL (Figures 7A–D). Given the small size of atherosclerotic plaques in the AS and BCA, it was impractical to extract mRNA for qRT-PCR, or total protein for Western blotting. We therefore used immunohistological methods to quantify the presence of M1 and M2 markers as well as selected MMPs and TIMP-3. Our *in vitro* studies together with the availability of suitable antibodies (Table 2) guided our choice of iNOS as suitable M1 marker and arg-1 and Ym-1 as suitable M2 markers. Based again on our *in vitro* studies and the availability of suitable antibodies (Table 2), we chose to study MMP-13 and MMP-14 as potentially related to classical activation. MMP-12 and TIMP-3 were also chosen for comparison because they are abundantly expressed *in vitro*, irrespective of classical and alternative activation. For each of the antibodies and lectin used the staining was specific, both in the aortic sinus (Figures 7A,B) and the BCA (Figures 7C,D) of either genotype. Interestingly, phenotypic markers, MMPs and TIMP-3 were mainly associated with GSL-positive areas rather than α-actin (Figures 7A–D). The percentage of the total plaque area stained with each antibody was measured using image analysis. By confining measurements to the plaque, we avoided any influence of staining from the media layer. Furthermore, some of the antibodies stained cardiac myocytes surrounding the aortic root (Figures 7A,B) but this did not distort our subsequent measurements because these areas were excluded from the quantification.

**Figure 8:**
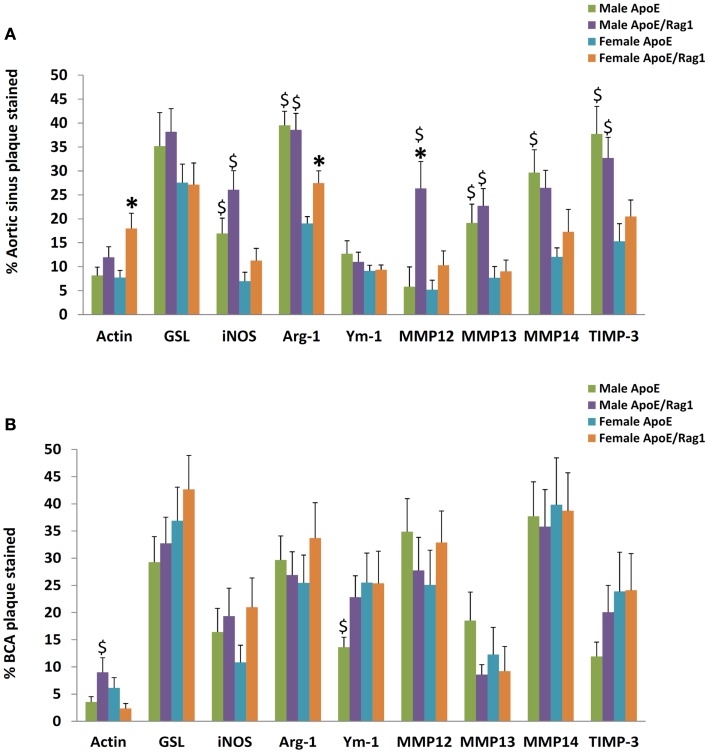
**The proportion of staining for cell type, M1/M2 markers, MMPs, and TIMPs in plaques from ApoE KO or ApoE/Rag1 DKO mice**. **(A)** Aortic sinus plaques. **(B)** Brachiocephalic artery plaques. Values are expressed as mean and SEM. *P < 0.05 vs. DKO, ^$^*P* < 0.05 indicates gender differences for that genotype, *n* = 11–19.

The area stained with α-SM actin in the aortic sinus (Figure 8A) was less than 20% under all conditions, consistent with the lipid-rich nature of plaques in this model at this time point. Nevertheless, SMC area was 1.5 times higher in the female DKO compared with ApoE KO mice (*P* = 0.0051) and the same trend was seen in the male mice. The pooled data for both genders were also significant (*P* = 0.0058) There were no differences between genotypes in the BCA (Figure 8B), although male DKO mice had 3.8-times more SMC staining than females (Figure 8B). Plaque areas stained with GSL were 30–40% in AS or BCA, consistent with highly inflamed nature of these plaques. GSL areas in the AS (Figure 8A) and BCA (Figure 8B) did not significantly differ in the two mouse genotypes or genders.

Quantitative measurements of staining for iNOS, arg-1, or Ym-1 showed that these areas were similar to or less extensive than GSL (Figures 8A,B), consistent with the concept of their being restricted to subpopulations of the GSL-positive cells (Figures 7A–D). Approximately, the same proportion of cells stained for iNOS and Ym-1, implying that approximately half of the FCMs had M1 or M2 markers, consistent with previous literature (12). Staining for arg-1 was more extensive and appeared to overlap with that for iNOS in some cells (Figures 7A–D and 8A,B). Staining for iNOS tended to be higher in DKO than ApoE KO mice in the aortic sinus (Figure 8A) and BCA (Figure 8B), although this was not significant. Based on this evidence, M1 activation of plaque FCMs occurs and may even be increased in the absence of T and B lymphocytes, irrespective of gender, and consistent with the PCR data on granuloma FCMs described above. Female DKO mice had 1.4-fold more arg-1 staining than ApoE KO mice in the aortic sinus (Figure 8A) and the same trend was seen in the BCA (Figure 8B). There were no differences in arg-1 staining for male DKO and ApoE KO mice at either site (Figures 8A,B). Staining for Ym-1 was also not different between genotypes in the aortic sinus (Figure 8A) or BCA (Figure 8B). Based on this evidence, M2 activation of FCM was present in the absence of T and B lymphocytes in mice of both sexes, and may be increased in the DKO females.

Additional gender differences were also noted. Staining for iNOS in the aortic sinus was 2.4 times greater in the male than female mice of both ApoE KO and DKO mice (Figure 8A). Staining for arg-1 was 2.1 and 1.4-fold higher in male compared with female ApoE and DKO mice, respectively in the aortic sinus (Figure 8A) although not the BCA (Figure 8B). Staining for Ym-1 was similar between males and females of either genotype in the aortic sinus, and in ApoE KO in the BCA (Figures 8A,B).

The areas stained for MMPs-12, -13, -14, and TIMP-3 were extensive in the aortic root and BCA plaques, which shows that these proteins are widely expressed in plaques (Figures 8A,B). The area of staining for MMP-13 and MMP-14 was similar in ApoE KO and DKO mice of either gender in the AS (Figure 8A) or BCA (Figure 8B). Clearly, the absence of T and B lymphocytes had little impact on extent of MMP-13 or MMP-14 staining, consistent with the PCR data obtained from granuloma FCMs. MMP-12, however, showed fourfold increased staining in male DKO compared to ApoE KO mice (*P* = 0.0261), although this was not replicated in the BCA (Figure 8B) or in female mice. The areas of TIMP-3 staining in the BCA were in all cases similar irrespective of genotype or gender (Figures 8A,B). Male mice of either genotype had approximately twice as much staining for MMP-13, MMP-14, or TIMP-3 than females in the AS (Figure 8A), although there was no difference in the BCA (Figure 8B).

## Discussion

The clear conclusion from our experiments is that FCMs in subcutaneous granulomas and atherosclerotic plaques acquired markers of classical (M1) and alternative (M2) activation even in the absence of lymphocytes. This conclusion was valid in male and female mice, despite differences in the size and composition of plaques between the two genders. Furthermore, since nuclear localized NF-κB, phospho-STAT-1 and phospho-STAT-6 were detected in granuloma FCMs, activators of the pathways leading to M1 and M2 polarization also existed in adequate quantities in the presence or absence of T or B lymphocytes. We also observed that expression of several MMPs and TIMPs occurred in granuloma and plaque FCMs *in vivo* independently of T and B lymphocytes.

Recent work demonstrated that foam cell formation did not, in itself, lead to M1 or M2 activation (28). By contrast, peritoneal FCM generated *in vivo* were resistant to M1 activation, owing to stimulation of the LXR pathway. Nevertheless, several studies showed that there are FCMs in mouse atherosclerotic plaques that express both M1 and M2 markers either *in situ* (12, 13) or after collagenase isolation (32). M1 and M2 markers were associated with distinct cell populations even though they had overlapping distributions in the intima of advanced mouse plaques (32). Our histological observations confirmed these findings but showed, in addition, that FCMs produced in the context of the foreign body reaction caused by a polyurethane sponge implantation also prominently expressed M1 and M2 markers. Granuloma FCMs mimicked the gene expression pattern of plaque FCMs and may therefore be useful as a more-easily isolated surrogate. Functionally, iNOS and arg-1 appear to compete for substrate. Indeed, in a previous study, we demonstrated this directly in rabbit FCMs where down-regulation of arg-1 compared to non-foamy macrophages led to decreased urea and increased nitrate release (24).

In previous papers dealing with atherosclerosis, it has been acknowledged that Th1- and Th2-lymphocyte related cytokines can polarize macrophages toward M1 and M2 phenotypes, respectively (11–13). This suggests that lymphocytes and hence, by implication, adaptive immunity plays an essential role in macrophage polarization, albeit in concert with other factors (11). On the contrary, our new data show clearly that M1/M2 polarization of FCMs can take place efficiently in the absence of T and B cells. To do this, we recreated a previously-characterized cross (20) between the well-established ApoE KO mouse and the equally well-characterized Rag1 KO mouse. We obtained similar development of plaques in these mice as previously observed (20) and then went on to make novel observations of macrophage phenotypes in subcutaneous granulomas and plaques.

It is worth noting that the absolute copy numbers of the M1 and M2 marker mRNAs measured in granuloma FCMs were of the same order of magnitude as those in measured in classical and alternatively activated macrophages *in vitro* (compare Table 5 to Figure 1A). Despite the obvious limitations of comparing data across such different experimental conditions, it is hard to escape the conclusion that the FCMs produced *in vivo* express M1 and M2 marker genes to a substantial degree, irrespective of the presence of lymphocytes. Moreover, the canonical pathways of classical activation, NF-κB and STAT-1, and alternative activation, STAT-6, are also triggered in T and B cell deficient mice. One possible explanation is that TLR-4 mediated induction of so-called interferon response factors (IRFs, specifically IRF-3 and IRF-7) can lead to the secretion of IFNα and IFNβ, thereby bypassing the requirement for IFNγ (9). Plaques contain several potential activators of TLRs (33); and these could well be the sources of classical activation. In addition to TLR agonists, other stimulators of the NF-κB pathway, including TNFα and IL-1, that are known to occur in mouse atherosclerotic plaques (3) could also act as classical activators independently of IFNγ, as shown in many previous studies (3, 14). The presence of these alternative mediators therefore provides a rationale for M1 activation in T and B cell depleted mice, although additional experiments beyond the present scope would be needed to identify the specific mediators. M2 polarization can also occur in response to a variety of mediators, although activation of STAT-6 appears to indicate the mediation of IL-4 and/or IL-13 in our mice even in the absence of lymphocytes. An additional, non-exclusive explanation for our findings is that there are sources other than lymphocytes for the cytokines associated with M1 and M2 activation in mice. For example, natural killer cells were shown to be an active source of IFNγ in Rag-1 KO mice (34) and could therefore account for the residual levels of IFNγ we observed in the blood of ApoE/Rag-1 DKO mice (Table 3). Likewise, mast cells (35) and neutrophils (36) are plausible sources of IL-4 and IL-13 in lymphocyte-depleted mice.

MMPs have been strongly implicated in the progression of atherosclerosis, and more particularly in ECM degradation as well migration, proliferation and apoptosis of vascular cells (5, 37). The results in Figure 1B showing that MMP-2, MMP-9, MMP-13, MMP-14, and MMP-25 were up-regulated and TIMP-2 down-regulated in mouse macrophages during classical activation are consistent with previously reviewed data for MMP-9 and MMP-13 (38). However, IFNγ had no effect on MMP or TIMP mRNA expression in mouse macrophages at the 18-h time point we used (Figures 1C,D), which was chosen to allow time for priming effects to be observed. Up-regulation of MMP-19 and down-regulation of MMP-14 were the only changes that we observed in response to IL-4 (Figure 1B). Furthermore, there was no difference in the expression level of any of the MMPs or TIMPs in FCMs isolated from sponge granulomas in ApoE KO and DKO mice. Despite this, there was a small increase in the proportion of cells able to degrade gelatin in DKO mice, which might be explained by the trend toward increases in MMP-2 and MMP-9 mRNA expressions. Further experiments would be needed to verify this. Recent work has placed increased emphasis on macrophage proliferation in mouse atherosclerosis (39). Specifically, it has been suggested that proliferation, rather than recruitment, is the major factor leading to accumulation of FCMs into atherosclerotic plaques of ApoE null mice at early time points (40). We found a relative decrease in FCMs from female DKO mice, but this did not appear to be associated with differences in M1/M2 polarization or MMP expression. Turning to our immunohistochemical studies of atherosclerotic plaques, few changes were noted in the extent of MMP-12, -13, -14, or TIMP-3 staining. Only MMP-12 staining appeared to be increased in male DKO mice in the AS but not BCA. Since, we found no effect of classical or alternative activation on MMP-12 expression *in vitro*, this isolated observation might be explained by another mediator such as GM-CSF, which has been shown to up-regulate MMP-12 in several, previously reviewed studies (38). However, the plasma cytokine levels (Table 3) provide no corroboration for this contention.

During the course of our studies, we noted significant differences in cytokine and lipid levels, plaque sizes, and content of SMC between male and female mice that obliged us to consider these data separately. We found smaller AS plaques in male than female ApoE KO mice, similar to what has been previously noted and attributed to the effects of estrogens (19) and prostaglandins (41). On the other hand, we found that males develop larger lesions in the BCA, confirming what we previously published in thesis form (29) and consonant with findings in the aorta at longer time points (18). This is most likely related, at least in part, to the higher total cholesterol and VLDL + LDL levels, we observed in male mice (Table 4). The important fact to stress, however, is that acquisition of M1 and M2 markers and expression of MMPs and TIMP was independent of T and B lymphocytes, irrespective of the gender of mice we analyzed.

Human atherosclerotic plaques also have prominent populations of FCMs that show M1 markers (42, 43) and have been known for many years to have nuclear localized NF-κB (44). Consistent with our present results, work on cells isolated from human atherosclerotic plaques, placed emphasis on innate immune mechanisms, by showing that TLR-2 activation plays an important role in M1 polarization and MMP secretion (45). There are also foci of FCMs in the intima that express M2 markers, which are distinct from FCMs carrying M1 markers (11). Non-foamy macrophages carrying M2 markers are even more prevalent in the adventitia (43). Hence, the distribution of cells carrying M2 markers in human plaques appears to be more restricted than in the mouse plaques in our study (Figures 7A–D) and in those previously published (12, 13).

With respect to MMPs and TIMPs, comparison of our *in vitro* results with published data from human macrophages isolated and incubated under very similar conditions (38, 42, 46) demonstrates many differences. For example, MMP-1 is absent but MMP-13 is abundant in mouse macrophages, whereas MMP-13 is absent and MMP-1 is abundant in human macrophages (42, 47), consistent with the limited distribution of MMP-13 in human tissues (48). Furthermore, MMP-12 is apparently much more abundant in mouse macrophages (Figure 1B) than human macrophages (42). Conversely, mRNAs for MMP-2, MMP-3, MMP-7, MMP-9, MMP-10, MMP-11, MMP-17, TIMP-1, and TIMP-3 appear much less abundant in mouse macrophages (Figure 1B) than human macrophages (42). Only MMP-8, MMP-14, MMP-19, MMP-25, and TIMP-2 show similar (within 10-fold) abundance in both species at the mRNA level. Responses of MMPs and TIMPs to classical and alternative also present stark contrasts between mice and men, under the conditions of our experiments. For example, expression of MMP-1, MMP-3, MMP-7, MMP-10, MMP-12, and TIMP-1 was increased by classical activation of human macrophages, whereas MMP-9 was constitutive (42), in contrast to what we observed here (Figure 1B). Furthermore, IFNγ increased MMP-1, MMP-10, MMP-12, and MMP-14 expression and decreased TIMP-3 expression in human macrophages (42), none of which we observed in mouse macrophages at the same time point (Figure 1C). Finally, IL-4 treatment increased MMP-11, MMP-12, and TIMP-3 expression in human macrophages (42) but MMP-19 expression in mice (Figure 1B). Similar disparity has been previously noted with respect to the expression levels of M1 and M2 markers in human and mouse macrophages (15). Moreover, the overall transcriptomic response to several *in vivo* models of inflammation appears highly divergent in mice and men (49). These limitations therefore caution against over-extrapolating our present results from a mouse model to human atherosclerosis.

In conclusion, our results definitively counter the hypothesis that lymphocytes are necessary for M1 or M2 polarization in mouse atherosclerosis, although more work will be needed to define the mediators responsible. Lymphocytes are also not needed for MMP and TIMP expression in FCMs *in vivo*. However, our studies do not rule out a modulatory role for T or B lymphocytes on either macrophage polarization or MMP production. It is conceivable that deletion of different lymphocyte populations has opposing effects of macrophage and foam cell activation, leading to a neutral effect overall. Subsequent studies using more selective interventions will be needed to investigate the role of specific lymphocyte subsets in mice.

## Conflict of Interest Statement

The authors declare that the research was conducted in the absence of any commercial or financial relationships that could be construed as a potential conflict of interest.
